# In Vitro and In Vivo Evaluation of a ^18^F-Labeled High Affinity NOTA Conjugated Bombesin Antagonist as a PET Ligand for GRPR-Targeted Tumor Imaging

**DOI:** 10.1371/journal.pone.0081932

**Published:** 2013-12-03

**Authors:** Zohreh Varasteh, Ola Åberg, Irina Velikyan, Gunnar Lindeberg, Jens Sörensen, Mats Larhed, Gunnar Antoni, Mattias Sandström, Vladimir Tolmachev, Anna Orlova

**Affiliations:** 1 Preclinical PET Platform, Department of Medicinal Chemistry, Faculty of Pharmacy, Uppsala University, Uppsala, Sweden; 2 Biomedical Radiation Sciences, Department of Radiology, Oncology and Radiation Sciences, Faculty of Medicine, Uppsala University, Uppsala, Sweden; 3 Organic Pharmaceutical Chemistry, Department of Medicinal Chemistry, Faculty of Pharmacy, Uppsala University, Uppsala, Sweden; 4 PET Centre, Centre for Medical Imaging, Uppsala University Hospital, Uppsala, Sweden; Wayne State University, United States of America

## Abstract

Expression of the gastrin-releasing peptide receptor (GRPR) in prostate cancer suggests that this receptor can be used as a potential molecular target to visualize and treat these tumors. We have previously investigated an antagonist analog of bombesin (D-Phe-Gln-Trp-Ala-Val-Gly-His-Sta-Leu-NH_2_, RM26) conjugated to 1,4,7-triazacyclononane-*N*,*N*',*N*''-triacetic acid (NOTA) via a diethylene glycol (PEG_2_) spacer (NOTA-P2-RM26) labeled with ^68^Ga and ^111^In. We found that this conjugate has favorable properties for *in vivo* imaging of GRPR-expression. The focus of this study was to develop a ^18^F-labelled PET agent to visualize GRPR. NOTA-P2-RM26 was labeled with ^18^F using aluminum-fluoride chelation. Stability, *in vitro* binding specificity and cellular processing tests were performed. The inhibition efficiency (IC_50_) of the [^nat^F]AlF-NOTA-P2-RM26 was compared to that of the ^nat^Ga-loaded peptide using ^125^I-Tyr^4^-BBN as the displacement radioligand. The pharmacokinetics and *in vivo* binding specificity of the compound were studied. NOTA-P2-RM26 was labeled with ^18^F within 1 h (60-65% decay corrected radiochemical yield, 55 GBq/µmol). The radiopeptide was stable in murine serum and showed high specific binding to PC-3 cells. [^nat^F]AlF-NOTA-P2-RM26 showed a low nanomolar inhibition efficiency (IC_50_=4.4±0.8 nM). The internalization rate of the tracer was low. Less than 14% of the cell-bound radioactivity was internalized after 4 h. The biodistribution of [^18^F]AlF-NOTA-P2-RM26 demonstrated rapid blood clearance, low liver uptake and low kidney retention. The tumor uptake at 3 h p.i. was 5.5±0.7 %ID/g, and the tumor-to-blood, -muscle and -bone ratios were 87±42, 159±47, 38±16, respectively. The uptake in tumors, pancreas and other GRPR-expressing organs was significantly reduced when excess amount of non-labeled peptide was co-injected. The low uptake in bone suggests a high *in vivo* stability of the Al-F bond. High contrast PET image was obtained 3 h p.i. The initial biological results suggest that [^18^F]AlF-NOTA-P2-RM26 is a promising candidate for PET imaging of GRPR *in vivo*.

## Introduction

Prostate cancer (PC) is the most frequently diagnosed non-cutaneous cancer and is the second cause of cancer-related mortality after lung and bronchus cancers in men [1]. An optimal PC treatment is guided by clinically staging the cancer, determining the extent of bone and soft tissue involvement, and selecting treatment options based on the stage. Different treatment modalities are used for organ-confined disease or PC beyond the confines of the prostate gland [2]. Although methods are available to visualize bone metastases [3], imaging of soft tissue metastases remains a significant problem for disease staging. Sensitive staging agents are urgently needed, especially to monitor soft tissue involvement. 

Cell membrane antigen targeting using radiolabeled peptides is a promising approach that can provide adequate staging of PC. Although prostate stem cell antigen (PSCA) and prostate-specific membrane antigen (PSMA) are expressed in primary prostate tumors and the vast majority of metastases, the natural ligands for the aforementioned antigens are unknown. The gastrin-releasing peptide receptor (GRPR) is an interesting alternative molecular target for visualization of PC that can be easily targeted by its natural ligand GRP or bombesin-like small peptides [4]. High GRPR-density expression in prostate carcinomas and prostatic intraepithelial neoplasms and the absence of receptor expression in normal prostate tissue and benign hyperplastic prostate tissue has previously been reported [5]. The data concerning the high levels of receptor expression in prostate tissues, which are in the earliest phase of the malignant transformation process, suggest that GRPR expression can be a marker of choice for the early detection of prostate carcinoma and local metastases [6]. 

Bombesin (BN) is a tetradecapeptide that binds to GRPR with high affinity. BN-agonists elicit a strong physiological response due to the activation of BN-receptors in the nervous system and the gut [7]. For instance, a DOTA-conjugated BN[7-14] agonist labeled with ^177^Lu (^177^Lu-AMBA) showed some side effects in a phase I dose escalation study [8]. The observed side effects for BN agonists have been hypothesized to be absent for antagonistic analogs [9,10,11]. BN-based antagonists were shown to have superior *in vivo* biodistribution and targeting properties to agonists [10].

**Figure 1:**
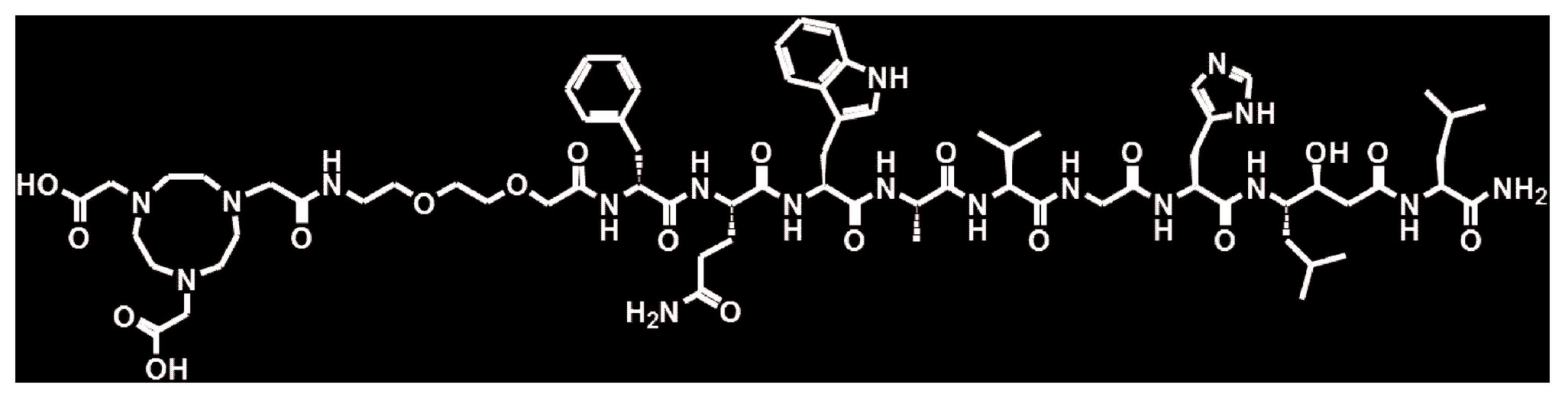
Structural formula of NOTA-PEG_2_-[D-Phe^6^,Sta^13^,Leu^14^]bombesin[6-14] (NOTA-P2-RM26).

Very recently, we reported data supporting the potential utility of a new radiolabeled BN-antagonist conjugate, NOTA-P2-RM26 (Figure **1**), to image GRPR-expressing tumors *in vivo* [12]. In this conjugate, the chelator NOTA (1,4,7-triazacyclononane-N,N',N''-triacetic acid) was coupled to D-Phe-Gln-Trp-Ala-Val-Gly-His-Sta-Leu-NH_2_ (RM26) [11,13] via diethylene glycol (PEG_2_) and labeled with radiometals: ^111^In was used for single photon emission computer tomography (SPECT), and ^68^Ga was used for positron emission tomography (PET) imaging. Fast clearance from the blood and receptor-positive organs together with high uptake and long retention in tumors led to increasing tumor-to-background ratios over time for this conjugate. 

Fluorine-18 is the most commonly used radioisotope for PET. The nuclear properties of ^18^F make it attractive as a label for peptide-based imaging agents. Its half-life (109.7 min) matches with the rapid pharmacokinetics of short peptides. Its low positron energy (E_β+,max_ = 0.64 MeV) results in a short positron range in tissues (theoretically calculated path length in water = 2.39 mm), making it well suited for high resolution PET images [14]. 

Until recently, the most common approach to the fluorination of peptides was a multistep synthesis of ^18^F-labeled precursors containing thiol-reactive malemides or primary amine-reactive succinimides and their coupling to peptides [15]. The conjugation was often non-regiospecific and generally resulted in low radiochemical yields [16]. Over the past several years, another convenient alternative for ^18^F-labeling, the silicon-fluoride acceptor (SiFA) approach, was developed. While the radiochemical yields are generally high, the increased overall lipophilicity of the peptides that were ^18^F-labeled via SiFA-radiochemistry resulted in an unfavorable biodistribution with high liver uptake and reduced bioavailability of tracers [17].

Recently, a new simple, one-step labeling method for the radiofluorination of peptides was reported by McBride et al. [18]. The method utilized the strength of the Al-F bond and the ability of NOTA to chelate aluminum. In many aspects this method resembles the labeling procedures for radiometals, such as ^68^Ga and ^111^In. The advantages of this approach are the relatively high yield, simplicity and robustness as well as the hydrophilic character of the label. 

The aim of this study was to evaluate a ^18^F-labeled competitive antagonistic analog of BN for PET imaging of GRPR expression in PC. To this end, NOTA-P2-RM26 was labeled with ^18^F via NOTA-AlF chelation chemistry. The labeling stability, *in vitro* binding specificity, inhibition efficiency and cellular processing of [^18^F]AlF-NOTA-P2-RM26 were investigated. Finally, the *in vivo* specificity and pharmacokinetics of [^18^F]AlF-NOTA-P2-RM26 were studied in NMRI and Balb/c nu/nu PC-3 tumor xenografted mice.

## Materials and Methods

The synthesis of NOTA-PEG2-[D-Phe6,Sta13,Leu14]bombesin[6-14] (further denoted as

NOTA-P2-RM26) with a molecular mass of 1543.8 Da has been previously reported [12]. Fluorine-18 was produced via the ^18^O(p,n)^18^F nuclear reaction using a Scanditronic MC-17 cyclotron (Uppsala, Sweden). A silver body target filled with 25% ^18^O-enriched water (Rotem) was used. High-performance liquid chromatography (HPLC) analysis was conducted on an Elite LaChrom system (Hitachi, VWR) consisting of an L-2130 pump, a UV detector (L-2400) and a radiation flow detector (Bioscan) coupled in series. Data acquisition and handling were performed using the EZChrom Elite Software Package. Sodium dodecyl sulfate polyacrylamide gel electrophoresis (SDS-PAGE) and instant thin-layer chromatography (ITLC) were used for analysis. The distribution of radioactivity along the ITLC strips and SDS-PAGE gels was measured on a Cyclone™ Storage Phosphor System (PerkinElmer). The radioactivity was measured in an automated ɤ-counter with a 3-inch NaI(Tl) detector (1480 WIZARD, WallacOy). The data were analyzed by an unpaired, two-tailed t-test using GraphPad Prism (version 4.00 for Windows GraphPad Software, San Diego, California, USA) to differentiate significant statistical differences (p<0.05).

### Radiolabeling and in vitro stability test

[^18^F]Fluoride (2-4 GBq) was trapped using a Chromafix 30-PS-HCO_3_ cartridge (Macherey-Nagel GMBH & Co.KG, Geramny). The cartridge was washed with 3 ml deionized water, and the ^18^F was eluted into siliconized Eppendorf tubes using a mixture of 0.462 M aq. NaCl:EtOH (25:75). Two hundred microliter fractions were collected. The second fraction, containing approximately 80% of the radioactivity (1-2 GBq), was buffered to pH 4.0 with 15 µl of 0.5 M sodium acetate (pH 4.0), followed by the addition of an aqueous solution of NOTA-P2-RM26 (25 µl, 25 nmol, Milli-Q water), 1.5 µl (15 nmol) AlCl_3_ in 0.1 M sodium acetate buffer (pH 4.0) and 100 µl EtOH. The reaction mixture was heated to 100°C for 20 min. The reaction mixture was then diluted with 3 ml of deionized water and passed through a 1 ml Oasis HLB cartridge (Waters). The cartridge was then washed with 5 ml of deionized water to remove any unreacted ^18^F. The radiolabeled product was eluted with 5×200 µl fractions of 1:1 EtOH/water. The chemical and radiochemical purity of [^18^F]AlF-NOTA-P2-RM26 was checked by ITLC (Biodex Medical Systems) using 0.2 M citric acid (pH 2.0) as the running buffer (Rf=0.0 for the peptide and Rf=1.0 for the free fluoride) and HPLC. The conditions were as follows: A=10 mM TFA; B=70% acetonitrile (MeCN), 30% H_2_O, and 10 mM TFA with UV-detection at 220 nm; gradient elution: 0-2 min at 35% B, 2-9 min at 35 to 100% B, 9-12 min at 100% B; and flow rate was 2.0 mL/min. The analytes were separated using an analytical column with a stationary phase consisting of covalently bonded pentylsilane (Discovery BIO Wide Pore C5; 5cm x 4.6 mm). The analyte was spiked with the original conjugate in order to confirm the identity.

The labeling stability was evaluated using SDS-PAGE analysis (200 V constant) on NuPAGE 4–12% bis-Tris gels (Invitrogen) with the aims to check the release of fluorine-18, transchelation of [^18^F]AlF to serum proteins, and cleavage of labeled protein by peptidases. Briefly, 5 nmol of [^18^F]AlF-NOTA-P2-RM26 was incubated in 0.5 ml of murine serum for 30 min at 37°C. After incubation, the samples were treated with NuPAGE® LDS Sample Buffer according to the manufacturer’s instructions and finally 0.05 nmol of sample loaded on the gel. Na^18^F-sodium fluoride was loaded in a separate lane as a low molecular weight internal reference.

To determine the IC_50_, NOTA-P2-RM26 was loaded with stable gallium and aluminum fluoride. The cold gallium loading procedure has been previously reported [12]. For fluorine loading, AlCl_3_ and NaF (both Sigma-Aldrich) were dissolved in Milli-Q water and mixed in equimolar amounts (30 nmol). The solution was buffered to pH 4.0 with 10 µl 0.5 M sodium acetate (pH 4.0) followed by the addition of NOTA-P2-RM26 (25 nmol, 25 µl in Milli-Q water) and 100 µl EtOH. The reaction mixture was heated to 100°C for 20 min. Both ^nat^Ga- and Al^nat^F-NOTA-P2-RM26 were analyzed by HPLC as described above. 

### In vitro studies

PC-3 human prostate cancer cells (ATCC, LGC Standards AB) expressing GRPR were cultured in RPMI media supplemented with 10% (v/v) FCS, 2 mM L-glutamine, and PEST (100 U/mL penicillin and 100 µg/mL streptomycin) (all from Biochrom AG). All *in vitro* experiments were performed in triplicate with 1×10^6^ cells/dish seeded one day before the experiment.

#### In vitro binding specificity assay

Two groups of cultured cell dishes were incubated with a [^18^F]AlF-NOTA-P2-RM26 solution (1 nM) for 1 h at 37 °C. One set of dishes was pre-saturated with a 1000-fold excess of unlabeled peptide that was added 5 minutes before the addition of [^18^F]AlF-NOTA-P2-RM26. After the incubation, the cells were washed and treated with 1 ml trypsin-ethylenediaminetetraacetic acid (EDTA) solution (0.25% trypsin, 0.02% EDTA in buffer; Biochrom AG). The detached cells were collected, and the cell-associated radioactivity was measured. The cell-bound radioactivity was expressed as a percentage of added radioactivity. 

#### In vitro competitive binding assay: IC_50_ determination

An *in vitro* competition experiment was performed using ^125^I-Tyr^4^-BBN (Perkin Elmer). Cell monolayers were incubated with ^nat^Ga- or Al^nat^F-NOTA-P2-RM26 (0 - 460 nM) in the presence of 0.1 pmol (100,000 cpm) ^125^I-Tyr^4^-BBN for 3 h at 4 °C. After the incubation, the cells were collected, and the cell-associated radioactivity was determined as described above. The IC_50_ values were determined using GraphPad software.

#### Cellular uptake and internalization assay

The cells were incubated with 2 nM of [^18^F]AlF-NOTA-P2-RM26 medium at 37 °C. At predetermined time points (1, 2, 3 and 4 h), the incubation medium was discarded, the cells were washed once and the membrane-bound and internalized radioactivity were collected using the method described earlier [12].

### In vivo studies

All animal studies were planned and performed in accordance with the national legislation on the protection of laboratory animals and the Ethic Committee for Animal Research of the Uppsala University approved the study plans. Randomly divided groups of 4 mice per data point were used in all experiments. The biodistribution of [^18^F]AlF-NOTA-P2-RM26 was evaluated in male NMRI mice (weight 37±3 g). BALB/c nu/nu male mice with PC-3 xenografts (weight 20±1 g at the time of the experiment) were used for tumor targeting and imaging studies. The tumors were grafted by subcutaneous injection of PC-3 cells (10^7^ cells/mouse) into the right hind legs 3 weeks before the experiment. The average tumor size was 0.42±0.05 g at the time of the experiment.

#### Biodistribution and in vivo binding specificity study in NMRI mice

NMRI male mice were intravenously injected with 45 pmol of [^18^F]AlF-NOTA-P2-RM26 (60 kBq in 100 µL of phosphate buffered saline (PBS)). One group of animals was co-injected with 20 nmol of unlabeled peptide to test the *in vivo* binding specificity of [^18^F]AlF-NOTA-P2-RM26 to murine GRPRs. The mice were euthanized 1 h post-injection (p.i.) by intraperitoneal administration of Ketalar-Rompun solution (10 mg/mL Ketalar and 1 mg/mL Rompun; 20 µL of solution per gram of body weight). Blood samples were collected by cardiac puncture. The organs of interest were collected, weighed and their radioactivity was measured. The organ uptake values were expressed as a percentage of the injected dose per gram of tissue (%ID/g) except for the gastrointestinal tract (GI) and carcass, which were expressed as %ID per whole sample.

#### Biodistribution and in vivo binding specificity studies in PC-3 tumor-bearing BALB/c nu/nu mice

PC-3 xenografted male BALB/c nu/nu mice were intravenously injected with 45 pmol of [^18^F]AlF-NOTA-P2-RM26 (100 µL in PBS). The mice were sacrificed at 1, 3 and 6 h p.i. to determine the uptake in tumors and other receptor-positive organs. A group of animals was co-injected with an excess of non-labeled peptide (20 nmol) and sacrificed 1 h p.i. The blood and other organs of interest were collected and treated as described above. 

### Imaging study

The experiment was performed to obtain a visual confirmation of the *ex vivo* biodistribution experiments. A PC-3 tumor-bearing mouse was sacrificed 3 h after injection of 45 pmol of [^18^F]AlF-NOTA-P2-RM26 (~ 2 MBq); the urinary bladder was excised; and the image was acquired using a clinical PET-CT scanner (GE Discovery VCT). The PET emission events were collected over 5 min. The image was acquired in 3-D mode and reconstructed in a 128×128 matrix with a 50-cm field of view (FOV).

## Results

### Radiolabeling and in vitro stability test

NOTA-P2-RM26 was successfully labeled with ^18^F. Within 1 h, a labeling yield of 60-65% (decay corrected) was achieved starting from 2.8 GBq Na^18^F (at end of bombardment). An effective specific activity of 55 GBq·μmol^-1^ was obtained.

**Figure 2:**
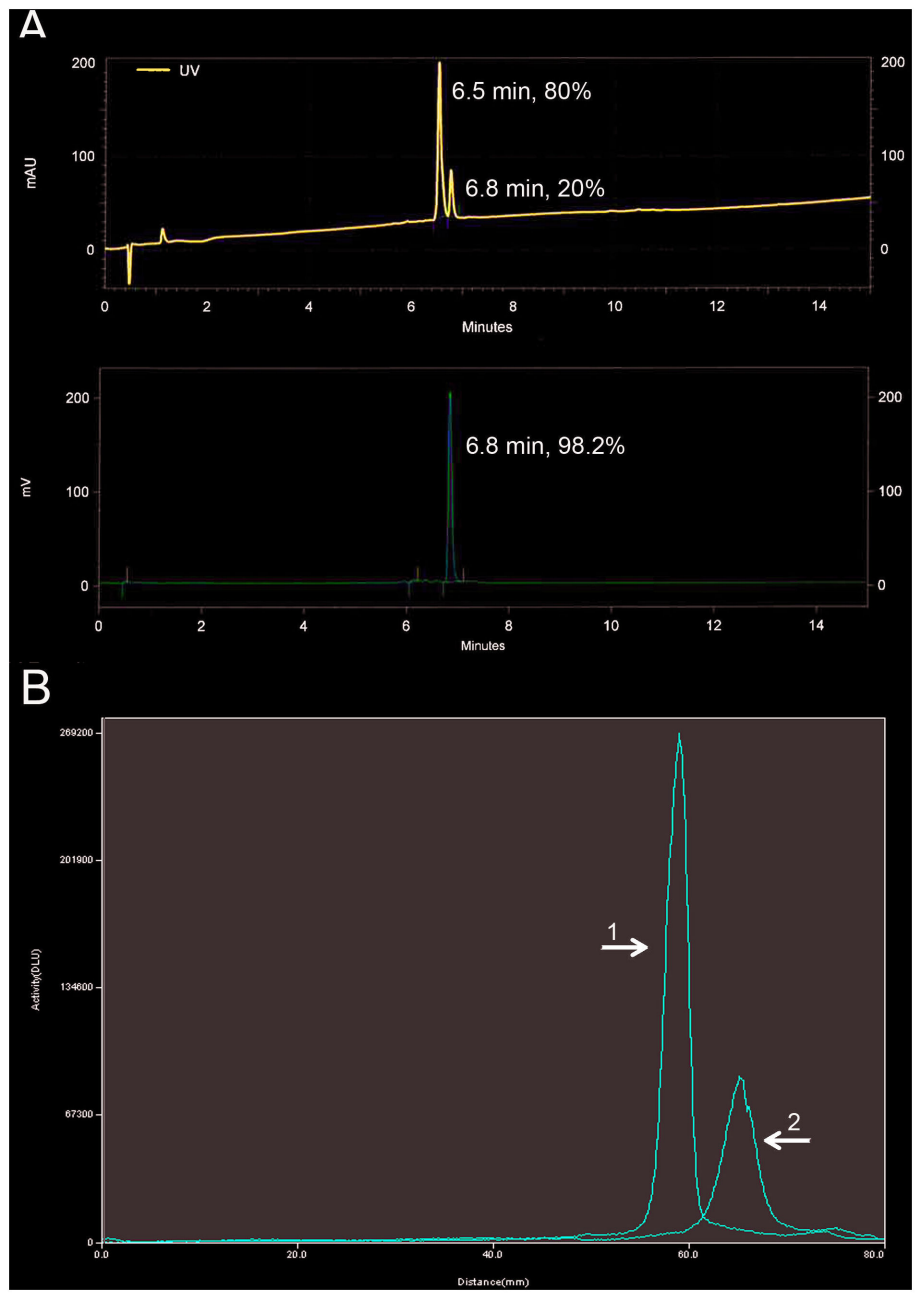
UV-radio-HPLC and SDS-PAGE analysis. **A**. UV-radio-HPLC analyses of [^18^F]AlF-NOTA-P2-RM26. Upper panel: UV profile with signals corresponding to the original conjugate, NOTA-P2-RM26 (6.5 min), and the product, [^18^F]AlF-NOTA-P2-RM26 (6.8 min). The recording wavelength was 220 nm. The analyte was spiked with the original conjugate in order to confirm the identity. Lower panel: Radioactivity profile with the principle signal (>98%) corresponding to the product, [^18^F]AlF-NOTA-P2-RM26. **B**. SDS-PAGE analysis of [^18^F]AlF-NOTA-P2-RM26 after a 1 h incubation in murine serum at 37°C (1). Radiolabeled sample (2). Na^18^F-sodium fluoride was used as low molecular weight radioactivity marker on the same gel.

The radiochemical purity exceeded 98% according to the HPLC analysis (Figure **2A**). SDS-PAGE analysis of the sample incubated in serum for 1 h at 37 °C showed a single radioactivity peak (Figure **2B**). No peak corresponding to free ^18^F was detected. 

### In vitro studies

#### In vitro binding specificity assay

**Figure 3:**
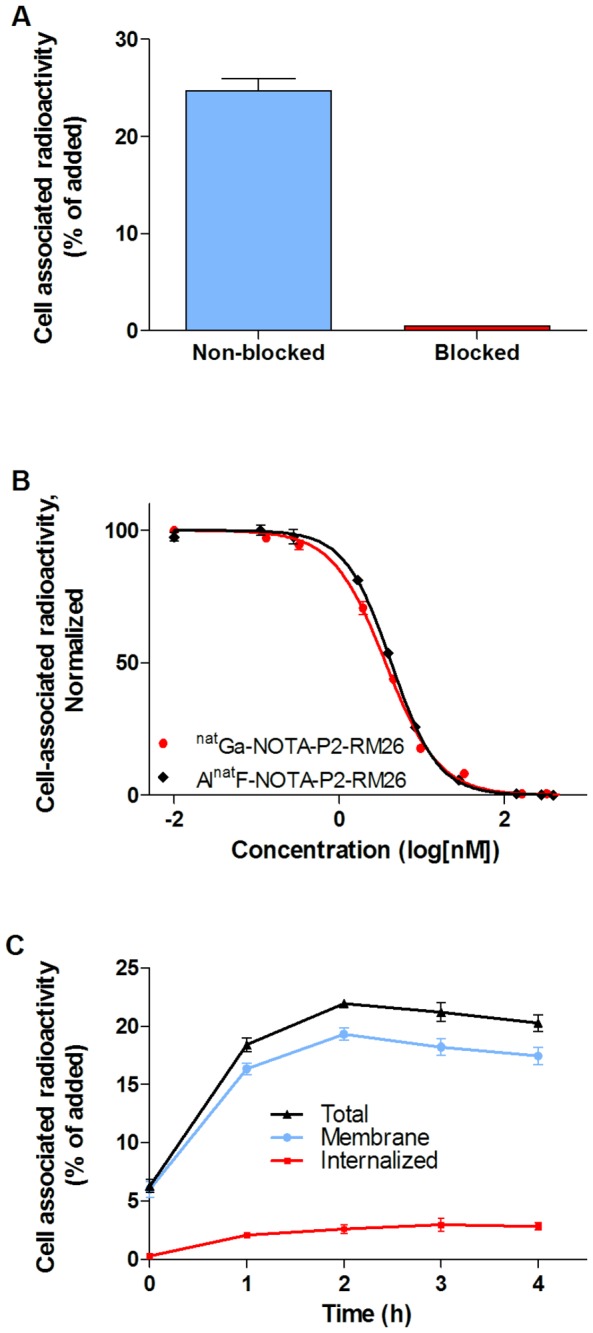
*In vitro* binding specificity, inhibition efficiency and cellular processing. **A**. *In* *vitro* binding specificity of [^18^F]AlF-NOTA-P2-RM26 to GRPRs was tested on PC-3 cells. Blocked dishes were pretreated with a 1000-fold excess of non-labeled peptide 5 min prior to the addition of 1 nM labeled compound. The cell-associated radioactivity is presented as a percentage of the total added radioactivity. **B**. Inhibition of ^125^I-Tyr^4^-BBN binding to PC3 cells with ^nat^Ga-NOTA-P2-RM26 or Al^nat^F-NOTA-P2-RM26. **C**. Cell-associated (internalized, membrane and total) radioactivity as a function of time after continuous incubation of PC-3 cells with [^18^F]AlF-NOTA-P2-RM26. Data are mean values ± SD of 3 culture dishes. Not all error bars are visible due to the small standard deviations.

To ensure that the GRPR-binding capacity of NOTA-P2-RM26 was preserved after labeling, the PC-3 cells were incubated with a [^18^F]AlF-labeled peptide. Binding of [^18^F]AlF-NOTA-P2-RM26 to the cells was specific as demonstrated by displacement by a non-radioactive peptide. A more than 57-fold decrease in radioactivity was observed for the cells pretreated with a 1000-fold excess of non-labeled peptide (Figure **3A**). 

#### In vitro competitive binding assay: IC_50_ determination

The binding properties of Al^nat^F-NOTA-P2-RM26 were compared to those of the ^nat^Ga-loaded sample in a competitive binding assay using ^125^I-Tyr^4^-BBN as the displacement radioligand (Figure **3B**). The IC_50_ values were in the same low nanomolar range with no significant difference (4.4±0.8 nM for [^nat^F]-AlF-NOTA-P2-RM26 and 3.5±0.5 nM for ^nat^Ga-NOTA-P2-RM26). 

#### Cellular uptake and internalization assay

The binding kinetics of [^18^F]AlF-NOTA-P2-RM26 to the GRPR were tested on PC-3 cells (Figure **3C**). The cellular uptake of [^18^F]AlF-NOTA-P2-RM26 reached a plateau within 2 h, when almost 22% of the added radioactivity was associated with the cells. The internalized radioactivity constituted less than 14% (13.9% ± 1.3%) of the cell-associated radioactivity after 4 h of continuous incubation of the cells at 37°C.

### In vivo studies

#### Biodistribution study in NMRI mice

**Figure 4:**
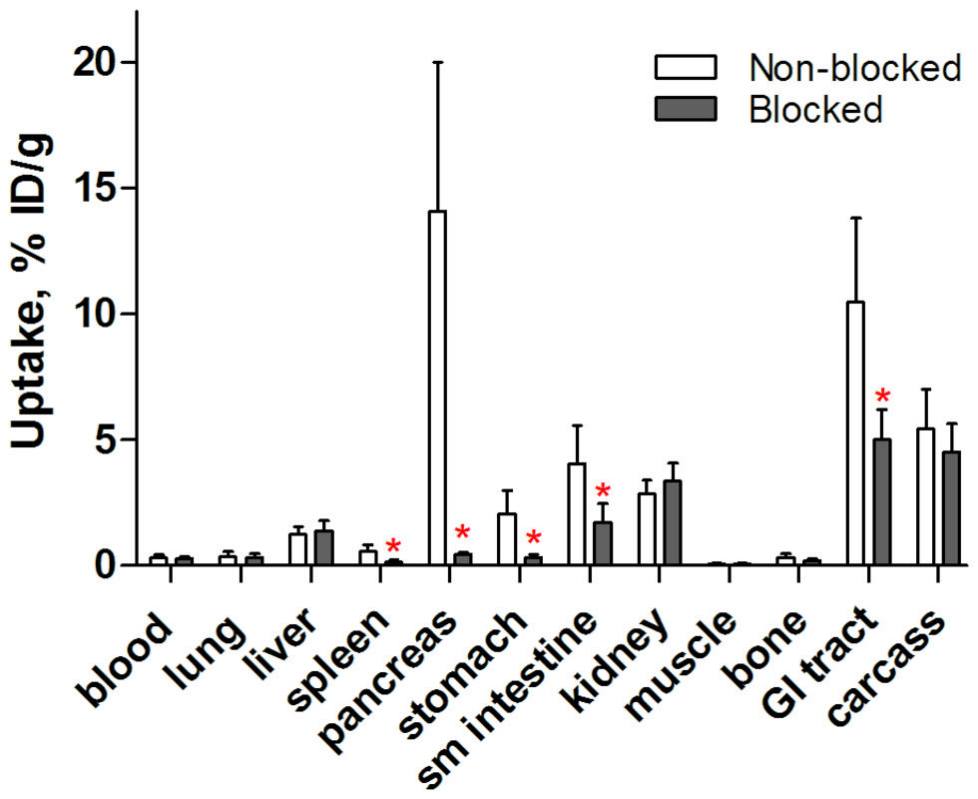
*In vivo* binding specificity in male NMRI mice. Biodistribution of [^18^F]AlF-NOTA-P2-RM26 in male NMRI mice 1 h p.i. The total injected mass of radiolabeled conjugate was 45 pmol, and all animals in the blocked group were co-injected with 20 nmol of the non-labeled peptide. The data are presented as the mean percentage of the injected dose per gram of tissue (%ID/g ± SD, n=4). The red asterisks denote significant differences between the groups injected with 45 pmol (non-blocked) and 20 nmol (blocked) (*p*<0.05).

Data concerning the biodistribution of [^18^F]AlF-NOTA-P2-RM26 in NMRI mice at 1 h p.i. are presented in Figure **4** and Table **S1**. The obtained data agreed well with a previous study that investigated the biodistribution of ^111^In and ^68^Ga-labeled NOTA-P2-RM26 [12]. Receptor-positive abdominal organs (pancreas, small intestine and stomach) showed high and specific uptake. The saturation of GRPR using a co-injection of non-labeled peptide significantly decreased the uptake of [^18^F]AlF-NOTA-P2-RM26 in these organs. [^18^F]AlF-NOTA-P2-RM26 also showed fast blood clearance via kidney excretion together with low kidney retention and liver uptake.

#### Biodistribution and in vivo binding specificity studies in PC-3 tumor-baring BALB/c nu/nu mice

**Figure 5:**
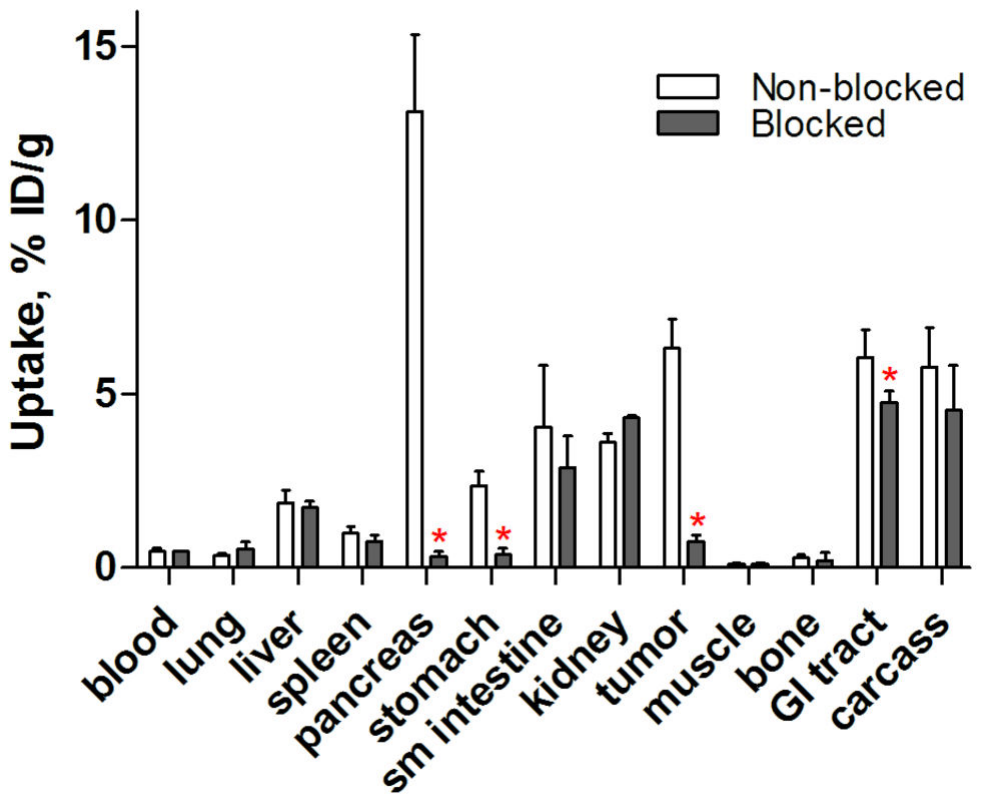
*In vivo* binding specificity in male Balb/c nu/nu mice bearing PC-3 xenografts. Biodistribution of [^18^F]AlF-NOTA-P2-RM26 in male Balb/c nu/nu mice bearing PC-3 xenografts 1 h p.i. The total injected mass of radiolabeled conjugate was 45 pmol, and the animals in the blocked group were co-injected with 20 nmol of the non-labeled peptide. The data are presented as the mean percentage of the injected dose per gram of tissue (%ID/g ± SD, n=4). The red asterisks denote significant differences between the groups injected with 45 pmol (non-blocked) and 20 nmol (blocked) (*p*<0.05).

**Figure 6:**
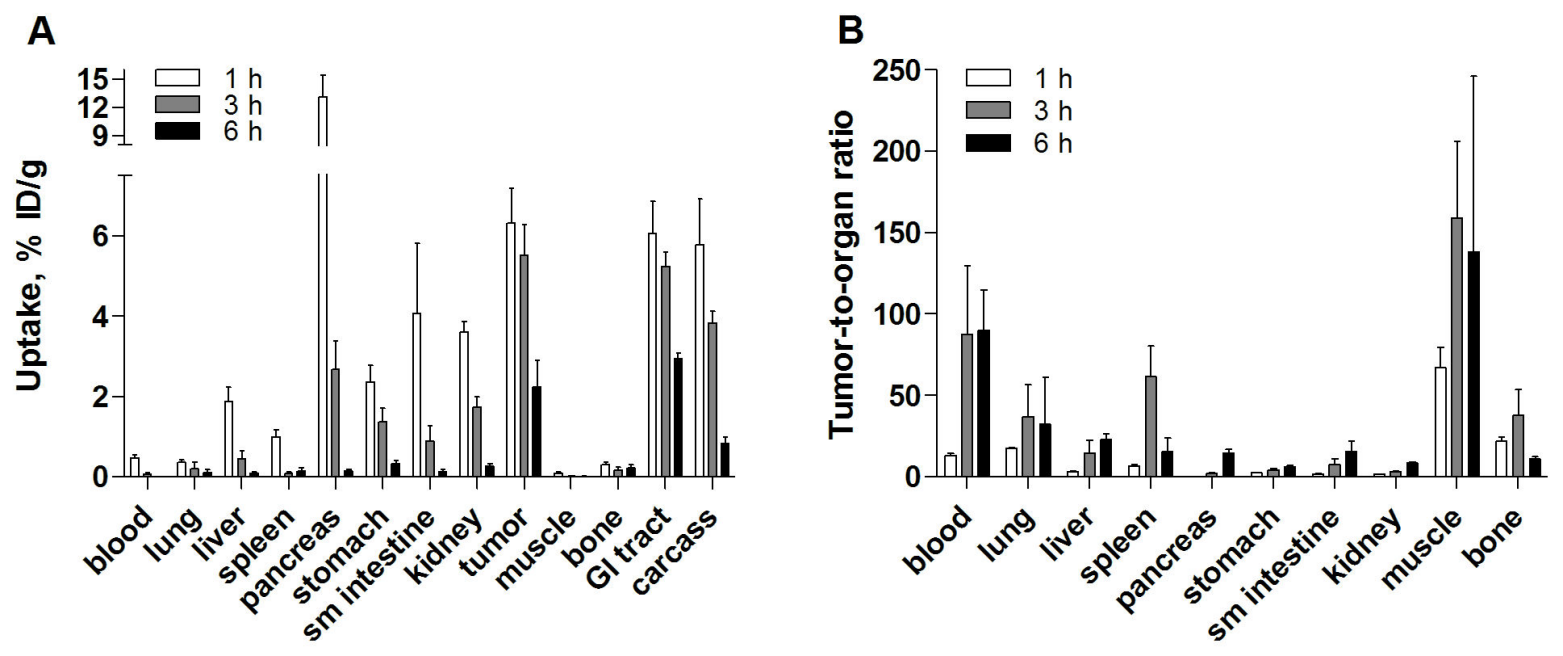
Biodistribution in male BALB/c nu/nu mice bearing PC-3 xenografts. **A**. Biodistribution of [^18^F]AlF-NOTA-P2-RM26 in male BALB/c nu/nu mice bearing PC-3 xenografts at different time points (total injected mass of 45 pmol). **B**. Tumor to normal organ ratios after injection of 45 pmol of [^18^F]AlF-NOTA-P2-RM26 in male BALB/c nu/nu mice bearing PC-3 xenografts. The data are presented as the mean percentage of the injected dose per gram of tissue (%ID/g ± SD, n=4).

The results of the biodistribution experiment in PC-3 xenografted mice are presented in Figure **5** and **6** and in Table **S2**. Xenografts and receptor-positive normal organs showed GRPR-specific uptake, which agreed with the data obtained from the NMRI mice. Pre-saturating the receptors with co-injection of non-labeled peptide decreased the tumor uptake more than 8-fold, from 6.3±0.9 %ID/g to 0.8±0.2 %ID/g (Figure **5**). Rapidly decreasing radioactive concentrations were observed in all receptor-expressing organs (e.g., pancreas uptake dropped from 13±2 %ID/g 1 h p.i. to 0.15±0.05 %ID/g 6 h p.i.) and the kidneys (from 3.6±0.3 %ID/g 1 h p.i. to 0.27±0.06 %ID/g 6 h p.i.) (Figure **6A**). The radioactive concentration in the tumors exceeded the radioactivity concentration in all organs and tissues 3 h p.i. (e.g., the tumor-to-blood ratio was 87±42 and the tumor-to-pancreas ratio was 2.2±0.6 3 h p.i.) (Figure **6B**). However, the radioactivity concentration in the tumors was stable between 1 and 3 h p.i. and then decreased more than 2-fold between 3 and 6 h p.i. High tumor-to-normal organ ratios were observed 3 h p.i.

### Imaging study

**Figure 7:**
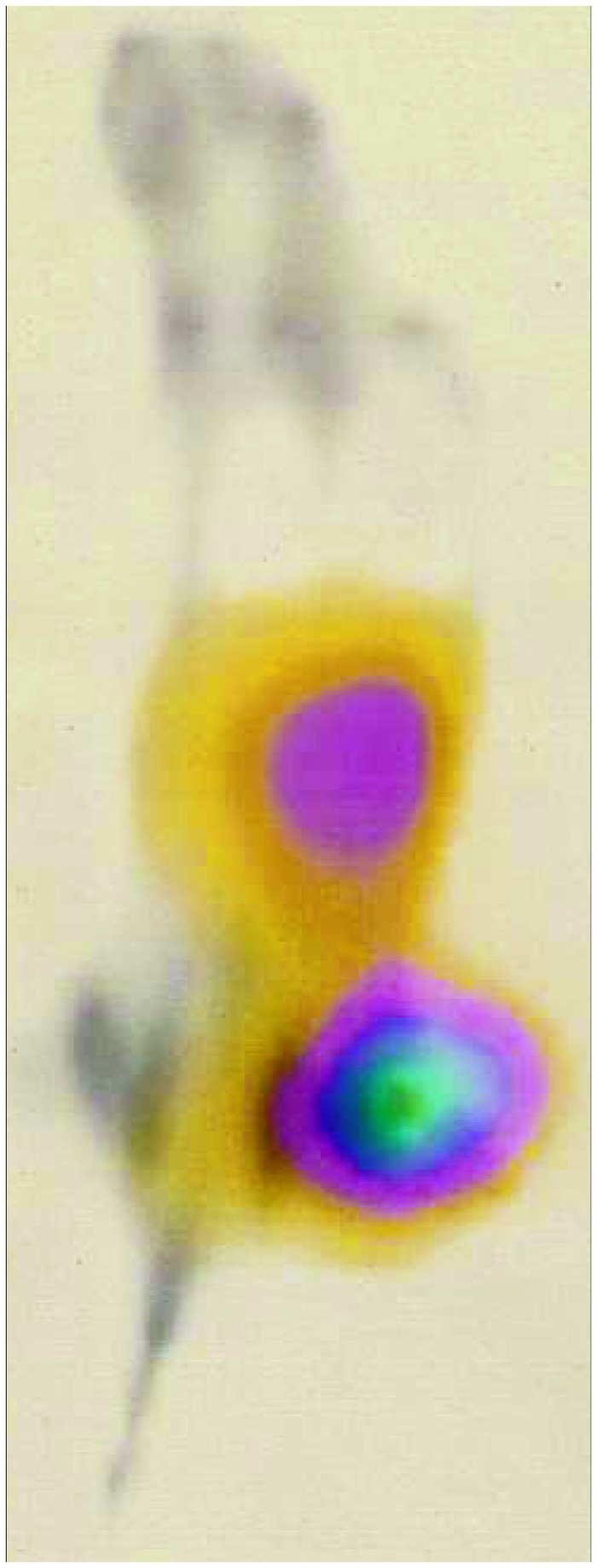
PET image. Imaging of GRPR expression in PC-3 xenografts in a BALB/c nu/nu male mouse. The animals was injected with 45 pmol of [^18^F]AlF-NOTA-P2-RM26 (~2 MBq). After 3 h, the animal was euthanized, the urinary bladder was dissected and the PET/CT images were acquired.

A mouse with PC-3 xenograft injected with fluorine-labeled peptide was imaged 3 h p.i. (Figure **7**). The tumor was clearly visualized, a finding that agreed well with the biodistribution data. Accumulation of radioactivity was also detected in the kidneys and abdominal area.

## Discussion

Detection of soft tissue metastases in PC is a challenge. Conventional anatomical imaging modalities, such as transrectal ultrasound (TRUS), computed tomography (CT) and magnetic resonance imaging (MRI), have demonstrated limited accuracy for the diagnosis of primary prostate tumors [19]. These methods have poor sensitivity, especially for soft tissue metastases, resulting in understaging and overtreatment of patients with extraprostatic disease [20]. Moreover, a variety of imaging agents in nuclear medicine which are used to visualize cellular metabolic activity ([^18^F]FDG, [^11^C]acetate, [^11^C]methionine) failed to detect PC soft tissue metastases due to low cancer cell metabolism [21,22,23]. [^11^C]/[^18^F]Choline uptake by tumors has also been studied. However, due to its moderate sensitivity and specificity, choline derivatives cannot be recommended as first-line imaging agents [19]. 

To be optimally useful in the current clinical context, a PET scan must be able to detect very small deposits of PC in the lymph nodes to guide therapy. 

A receptor-mediated tumor-targeting concept might show a higher specificity for the site of action. [^18^F]fluorodihydrotestosterone (FDHT)-based imaging was evaluated in prostatic carcinoma with increased androgen receptor (AR) expression. It is used to assess the receptor status and quantify changes in the receptors. However, the oncogenic changes in the AR in PC patients over time may affect the specificity of the ligand [24]. Until recently, only ^111^In-labeled full-length monoclonal antibody (mAb) capromab pendetide (ProstaScint) was available to image prostate-specific membrane antigen (PSMA). This antibody is directed against an epitope on the cytoplasmic domain of PSMA. However, the sensitivity and specificity of this imaging agent is far from optimal [24]. The labeling of two anti-PSMA mAbs (7E11 and J591, targeting the intra- and extra- cellular domains of PSMA, respectively) with ^89^Zr [25,26] has recently been reported. In a murine xenograft model, both mAbs demonstrated high and specific tumor uptake (up to 40%ID/g at 4 d p.i.) as well as high liver, spleen and bone uptake. The slow blood clearance of antibodies is also a limitation of this class of proteins as imaging probes. PSMA inhibitors based on glutamate-urea-lysine analogues have recently been developed [27]. Two derivatives, ^123^I-MIP-1072 and ^123^I-MIP-1095, were tested in a Phase 1 study of men and were capable of detecting lesions in soft tissue, bone, and the prostate gland as early as 1-4 h after injection [28]. Other radiolabeled small molecule PSMA inhibitors are under evaluation for PET and SPECT imaging of PC [29].

Because GRPR is expressed in 63-100% of primary prostate tumors and over 50% of metastases, it presents an attractive target for both therapeutic and diagnostic applications [30]. The GRPR expression level in human prostate tumors differs over time. Its expression is markedly upregulated in early-stage tumors, while lower expression has been reported in progressive prostate tumors [6]. This expression pattern makes GRPR useful as a prognostic biomarker. Prostate cancers with a higher GRPR density showed a better prognosis than ones with low or negative expression [31]. Development of PET tracers for GRPR expression could potentially add prognostic value by receptor quantification.

A recent paradigm shift toward G protein-coupled receptor antagonists after sst_2_ and sst_3_-targeting with somatostatin analogs has been observed [32]. Bombesin antagonists also showed superior targeting properties compared to GRPR agonists. High and sustained tumor uptake of Demobesin 1 increased the appreciation of the radiolabeled bombesin antagonists used as an imaging agent [10]. The potential capacity of the bombesin antagonist NOTA-P2-RM26 as an imaging agent for GRPR-expressing tumors has been verified in our previous work [12]. In the present work, the use of ^18^F-labeled NOTA-P2-RM26 to image GRPR with PET was investigated. NOTA-P2-RM26 was stably radiolabeled with ^18^F using a chelator-based labeling method, AlF-chemistry [18], with reasonably high yield within 1 h and a high radiochemical purity after a simple cartridge purification. The obtained specific radioactivity for [^18^F]AlF-NOTA-P2-RM26 was in the range of reported for other BN-antagonists [9,33] which corresponds to approximately 5.8 GBq injected radioactivity into human if protein dose per body weight would be the same as in this study. SDS-PAGE analysis suggests that fluorine-18 is not released, the radiopeptide is not cleaved by peptidases and [^18^F]AlF does not undergo transchelation to serum proteins. 

The *in vitro* characteristics of [^18^F]AlF-NOTA-P2-RM26 were investigated and it was found that the radiolabeled conjugate bound specifically to human GRPRs.

The binding properties of [^nat^F]AlF-NOTA-P2-RM26 did not differ from those of the ^nat^Ga-loaded peptide. Both [^nat^F]AlF- and ^nat^Ga-NOTA-P2-RM26 showed similar low nanomolar affinity in a competitive binding assay. In accordance with the data for ^111^In- and ^68^Ga-labeled peptides from our previous work [12], the PC-3 cells slowly internalized [^18^F]AlF-NOTA-P2-RM26 in the cellular processing experiment. This finding indicates that the antagonistic function of RM26 is preserved after modification by radiolabeling. 

Overall, the biodistribution of [^18^F]AlF-NOTA-P2-RM26 was similar to that of ^111^In- and ^68^Ga-labeled NOTA-P2-RM26 [12]. The biodistribution of [^18^F]AlF-NOTA-P2-RM26 in normal mice demonstrated specific uptake in receptor-positive organs (e.g., pancreas, stomach and small intestine) that was significantly decreased by the co-injection of an excess amount of non-labeled peptide. This finding suggests that the peptide cross-reacts with murine GRPRs. The tracer showed also specific tumor uptake in PC-3 xenografts. The peptide was mainly excreted via the kidneys, and low liver and gastrointestinal tract (with content) uptake rates were observed. A fast excretion and low degree of renal re-absorption was found. The rapid blood clearance observed for this conjugate agreed well with the ^111^In- and ^68^Ga-NOTA-P2-RM26 data [12]. Low uptake by the bones indicates a high *in vivo* stability of Al-F bond in [^18^F]AlF-NOTA-P2-RM26. The same pharmacokinetic behavior of rapid radioactivity release from normal GRPR-positive organs that was observed for ^111^In/^68^Ga-NOTA-P2-RM26 [12] was also found for [^18^F]AlF-NOTA-P2-RM26. In contrast to previous data, the washout of [^18^F]AlF-NOTA-P2-RM26 from PC-3 tumors was faster than that of ^111^In-NOTA-P2-RM26 [12]. The tumor uptake for [^18^F]AlF-NOTA-P2-RM26 decreased by more than 2.4-fold between 3 and 6 h p.i., while a less than 1.3-fold drop was observed previously for ^111^In-NOTA-P2-RM26 uptake in PC-3 xenografts between 3 and 24 h p.i. [12]. The data suggest that the optimal imaging time point for this tracer would be 3 h p.i., at which time the tumor-to-normal organ ratios are maximized. The potential of ^18^F-labeled conjugate for visualization of GRPR-positive tumors was confirmed by an additional imaging study. Images obtained with high contrast 3 h after the injection of [^18^F]AlF-NOTA-P2-RM26 clearly visualized GRPR-expressing PC-3 xenografts. However, the accumulation of radioactivity in the kidneys and abdominal organs was appreciably lower for [^68^Ga]-NOTA-P2-RM26, which resulted in higher image contrast (Table **1**) [12]. Still, because of the wide availability and the superior imaging characteristics of ^18^F, [^18^F]AlF-NOTA-P2-RM26 could be a suitable alternative to [^68^Ga]-NOTA-P2-RM26 for clinical PET imaging. 

**Table 1:** Biodistribution of [^18^F]AlF-NOTA-P2-RM26, [^111^In]-NOTA-P2-RM26 and [^68^Ga]-NOTA-P2-RM26 in male BALB/c nu/nu mice with PC-3 xenografts, 3 h p.i.

Organ	[^18^F]AlF-NOTA-P2-RM26	[^111^In]-NOTA-P2-RM26	[^68^Ga]-NOTA-P2-RM26
Blood	0.08±0.03	0.10±0.02	0.32±0.05
Lung	0.21±0.17	0.29±0.05	0.70±0.20
Liver	0.45±0.21	1.53±0.21	1.71±0.13
Spleen	0.10±0.03	0.41±0.08	0.87±0.11
Pancreas	2.7±0.7	3.8±1.1	0.66±0.26
Stomach	1.37±0.35	0.90±0.08	0.56±0.15
Small intestine	0.88±0.39	0.95±0.22	0.27±0.06
Kidney	1.74±0.25	2.9±0.5	1.37±0.17
Tumor	5.53±0.75	5.8±1.3	7.4±1.4
Muscle	0.03±0.01	0.06±0.01	0.03±0.01
Bone	0.17±0.08	0.17±0.08	0.25±0.08

Total injected mass of radiolabeled conjugate was 45 pmol. The data are presented as the mean percentage of the injected dose per gram of tissue (%ID/g ± SD, n=4).

## Conclusions

[^18^F]AlF-NOTA-P2-RM26 exhibited a high affinity for GRPRs, demonstrated specific targeting to GRPRs both *in vitro* and *in vivo* and showed favorable *in vivo* pharmacokinetics in normal and tumor-xenografted mice. An ^18^F-labeled tracer could be a suitable alternative for high-resolution PET imaging to stage PC and quantify GRPRs.

## Supporting Information

Table S1 **Biodistribution of [^18^F]AlF-NOTA-P2-RM26 in male NMRI mice 1 h p.i.**
(DOCX)

Table S2 **Biodistribution of [^18^F]AlF-NOTA-P2-RM26 in male BALB/c nu/nu mice with PC-3 xenografts.**
(DOCX)
