# Supplementary material for: In Vitro and In Vivo Evaluation of a 18F-Labeled High Affinity NOTA Conjugated Bombesin Antagonist as a PET Ligand for GRPR-Targeted Tumor Imaging
Source: PLoS One. 2013 Dec 3;8(12):e81932. doi: 10.1371/journal.pone.0081932 (PMC3849266; doi:10.1371/journal.pone.0081932)
Supplement: Table S1 — Biodistribution of [18F]AlF-NOTA-P2-RM26 in male NMRI mice 1 h p.i. (DOCX) [file pone.0081932.s001.docx]

**Table S1.** Biodistribution of [^18^F]AlF-NOTA-P2-RM26 in male NMRI mice 1 h p.i.

| **Organ** | **1 h** | **1 h Blocked** |
| --- | --- | --- |
| **Blood** | 0.3±0.1 | 0.3±0.1 |
| **Lung** | 0.3±0.2 | 0.3±0.1 |
| **Liver** | 1.2±0.3 | 1.4±0.4 |
| **Spleen** | 0.6±0.3 | 0.16±0.08* |
| **Pancreas** | 14±6 | 0.4±0.1* |
| **Stomach** | 2±1 | 0.3±0.1* |
| **Small intestine** | 4±2 | 1.7±0.7* |
| **Kidney** | 2.8±0.5 | 3.4±0.8 |
| **Muscle** | 0.09±0.03 | 0.08±0.03 |
| **Bone** | 0.3±0.2 | 0.2±0.1 |
| **GI tract** | 11±3 | 5±1 |
| **Carcass** | 5±2 | 5±1* |

The total injected mass of radiolabeled conjugate was 45 pmol, and the animals in the blocked group were co-injected with 20 nmol of the non-labeled peptide). The data are presented as the mean percentage of the injected dose per gram of tissue (%ID/g ± SD, n=4). The asterisks denote significant differences between the blocked and non-blocked animals (*p*<0.05).
