# Supplementary material for: In Vitro and In Vivo Evaluation of a 18F-Labeled High Affinity NOTA Conjugated Bombesin Antagonist as a PET Ligand for GRPR-Targeted Tumor Imaging
Source: PLoS One. 2013 Dec 3;8(12):e81932. doi: 10.1371/journal.pone.0081932 (PMC3849266; doi:10.1371/journal.pone.0081932)
Supplement: Table S2 — Biodistribution of [18F]AlF-NOTA-P2-RM26 in male BALB/c nu/nu mice with PC-3 xenografts. (DOCX) [file pone.0081932.s002.docx]

**Table S2**. Biodistribution of [^18^F]AlF-NOTA-P2-RM26 in male BALB/c nu/nu mice with PC-3 xenografts.

| **Organ** | **1 h** | **1 h Blocked** | **3 h** | **6 h** |
| --- | --- | --- | --- | --- |
| **Blood** | 0.48±0.08 | 0.4±0.2 | 0.08±0.03 | 0.023±0.001 |
| **Lung** | 0.36±0.06 | 0.4±0.3 | 0.2±0.2 | 0.11±0.08 |
| **Liver** | 1.9±0.4 | 1.6±0.3 | 0.5±0.2 | 0.10±0.02 |
| **Spleen** | 1.0±0.2 | 0.7±0.2 | 0.10±0.03 | 0.15±0.08 |
| **Pancreas** | 13.1±0.2 | 0.3±0.2* | 2.7±0.7 | 0.15±0.05 |
| **Stomach** | 2.4±0.4 | 0.4±0.1* | 1.4±0.4 | 0.33±0.08 |
| **Small intestine** | 4±2 | 2.6±0.9 | 0.9±0.4 | 0.13±0.06 |
| **Kidney** | 3.6±0.3 | 3.9±0.8 | 1.7±0.3 | 0.27±0.06 |
| **Tumor** | 6.3±0.9 | 0.8±0.2* | 5.5±0.8 | 2.3±0.7 |
| **Muscle** | 0.10±0.03 | 0.09±0.04 | 0.03±0.01 | 0.02±0.01 |
| **Bone** | 0.30±0.08 | 0.2±0.2 | 0.17±0.08 | 0.22±0.08 |
| **GI tract** | 6.1±0.8 | 4.8±0.3* | 5.2±0.4 | 3.0±0.1 |
| **Carcass** | 6±1 | 4±1 | 3.8±0.3 | 0.8±0.2 |

| **Tumor-to-Organ** | **1 h** | **3 h** | **6 h** |
| --- | --- | --- | --- |
| **Blood** | 13.27±1.24 | 87±42 | 89±25 |
| **Lung** | 17.4±0.8 | 36±20 | 32.5±28.8 |
| **Liver** | 3.4±0.2 | 14±8 | 23±3 |
| **Spleen** | 6.5±1.2 | 61±19 | 15.5±8.3 |
| **Pancreas** | 0.49±0.04 | 2.2±0.6 | 14.7±2.3 |
| **Stomach** | 2.7±0.1 | 4.25±1.16 | 6.4±0.6 |
| **Small intestine** | 1.7±0.6 | 7.4±3.7 | 15.5±6.5 |
| **Kidney** | 1.74±0.16 | 3.2±0.6 | 8.4±0.8 |
| **Muscle** | 66.9±12.8 | 159±47 | 138±108 |
| **Bone** | 21.7±2.8 | 38±16 | 10.8±1.7 |

The total injected mass of radiolabeled conjugate was 45 pmol, and the animals in the blocked group were co-injected with 20 nmol of non-labeled peptide. The data are presented as the mean percentage of the injected dose per gram of tissue (%ID/g ± SD, n=4). The asterisks denote significant differences between the blocked and non-blocked animals 1 h p.i (*p*<0.05).
